# The Role of Imaging Investigations in Evaluation of Cochlear Dimensions in Candidates for Cochlear Implantation—Our Experience

**DOI:** 10.3390/medicina59122086

**Published:** 2023-11-27

**Authors:** Cristian Mircea Neagoș, Eugenia Maria Domuța, Anca Gabriela Vlad, Adriana Neagoș

**Affiliations:** 1 Department of Otorhinolaryngology, Emergency County Hospital of Targu Mures, George Emil Palade University of Medicine Pharmacy Science and Technology, 540067 Targu Mures, Romania; neagos.cristian-mircea.23@stud.umfst.ro; 2 Department of Otorhinolaryngology, Faculty of Medicine and Pharmacy, University of Oradea, 410087 Oradea, Romania; maria.domuta@yahoo.com; 3 Intensive Care Department, Emergency County Hospital of Targu Mures, 540136 Targu Mures, Romania

**Keywords:** cochlear implant, computer tomography, cochlea width, cochlea length

## Abstract

*Background and Objectives*: The Cochlear implant is the first approved cranial nerve stimulator that works by directly stimulating the cochlear nerve. Various attempts have been made to evaluate the dimensions of the cochlea related to cochlear implantation. The preoperative computed tomographic examination is essential not only in assessing the anatomical aspect of the cochlea, but also in determining its dimensions to choose an appropriate electrode and obtain the best possible audiological performance. *Materials and Methods:* In the present paper, we aimed to carry out an observational study regarding the role of cochlear measurements in the preoperative evaluation of patients proposed for cochlear implants. The purpose of the study was to measure the cochlea and establish the existence of a correlation between the size of the cochlea and the age and gender of the patients. *Results:* From the group of 35 examined patients, 54% (n = 19) were male and 46% (n = 16) were female. The average length of the cochlea in the age group 0–4 years is 7.82 mm in the left ear and 7.86 mm in the right ear; in the age group 4–7 years, it is 7.82 mm and 7.94 mm, respectively; for the age group 7–14 years, the dimensions increase to 8.48 mm and 8.77 mm, respectively; and after 14 years, these dimensions reach 9.12 mm and 9.18 mm, respectively. Comparative measurements of the length of the cochlea by age groups show an increase in length with the patient’s age, but this increase does not exceed 1.5 mm for both the right and left ears. The measurements of the width of the cochlea, by age group, start from 6.84 mm in the left ear and 6.81 mm in the right ear at 0–4 years, 6.94 mm and 6.97 mm, respectively, in the group 4–7 years, 7.71 mm and 7.55 mm at 7–14 years, and reaching 8.19 mm and 8.12 mm at the age of 14 years and over. *Conclusions:* From the study carried out, it can be concluded that the evaluation of the dimensions of the cochlea is important for cochlear implantation. The size variables, although small, are still an element to be considered in correlation with the age of the patient and the implanted ear. This increase is statistically insignificant, but it still exists, even if, from a theoretical point of view, it is considered that the dimensions of the cochlea remain constant.

## 1. Introduction

The cochlear implant is the first example of a neural prosthesis that can replace a sensory organ, overcoming the non-functional auditory periphery of people suffering from bilateral sensorineural hearing loss. The cochlear implant is the first approved cranial nerve stimulator that works by directly stimulating the cochlear nerve. The medical and societal impact of this revolutionary device cannot be understated [1]. The cochlear implant is a neural prosthesis that can replace a sensory organ by restarting an electrical stimulation of the cochlear nerve. With the help of this cochlear amplifier called a cochlear implant, low-amplitude waves are transformed into high-amplitude [1]. Cochlear implants have a long history, complete with innovations that have resulted in a high-performing device today. The devices are totally implanted, improving neural health and survival through drug-directed therapy, intraneural electrode placement, electroacoustic stimulation, and hybrid cochlear implants, and methods to improve the neural prosthetic interface are evolving areas of cochlear implant innovation.

The temporal bone has a very complex anatomical structure in which the sensory organs of the cochlea and the vestibular system are contained in a small space, together with the sound conduction system of the middle ear.

The bony labyrinth consists of a system of semicircular canals, represented by the cochlea and the vestibule. The cochlear canal lies within the bony labyrinth, being attached to the internal and external walls. Thus, two separate spaces are created, represented by the scale tympany and the scale vestibuli, which communicate with each other at the tip of the cochlea through the helicotrema. It is also flooded with perilymph; for this reason, vibrations can only be effectively transmitted if there is a simultaneous pressure adjustment. This occurs at the level of the round window, located below the oval window at the end of the tympanic space, and is covered by a movable membrane.

The cochlea consists of a coiled labyrinth like a snail that is about 10 mm high and about 2.5 turns in humans embedded in the temporal bone of the skull. The cochlea has three cavities, represented by the cochlear duct, the scale vestibuli, and the tympani scale. The vestibuli scale is in the upper part containing the perilymph, which is separated from the median scale by a flexible partition called Reissner’s membrane at the level of which a potential difference occurs, and this is separated from the tympanic scale in the lower part, which continues inferiorly to the helicotrema to the round window through a rigid partition that includes a more flexible section called the basilar membrane.

Therefore, detailed imaging is necessary in this anatomical area. Imaging of the cochlear region is one of the most important preoperative aspects, defining the etiology of the hearing loss, locating anomalies that contraindicate surgery, selecting the ear to be implanted, evaluating the anatomy for surgery, and as far as possible, identifying potential complications. Thus, the ideal evaluation contains both high-resolution computed tomography and nuclear magnetic resonance of the temporal bone and central nervous system. The value of computed tomography and nuclear magnetic resonance in the routine evaluation of candidates for cochlear implantation varies by patient, although the performance of both investigations is mandatory. Due to the high costs and inherent risks, both investigations are not performed in every clinical situation. In general, the results of both investigations must be correlated with the particular intraoperative anatomical situations. Decreasing unnecessary preoperative imaging can potentially decrease the cost of cochlear implantation. In this sample, preoperative imaging did not affect the surgeon’s choice of which side to operate on. However, imaging may provide an anatomic roadmap and contribute to either surgical confidence or caution. With the increasing amount of cochlear implant-eligible elderly adults, preoperative imaging needs to be more clearly defined in this unique population [2].

There are many clinical objectives for which the highest possible spatial resolution is required. These include locating cerebrospinal fluid (CSF) fistulas, detecting malformations of the middle and inner ear, vestibulocochlear nerve, and facial nerve, and locating abnormalities of arterial and venous structures while also confirming dehiscence of the semicircular canals. The cochlea, from an anatomical point of view, can vary in size, however, the cochlear structures remain approximately similar. However, there are certain changes in size, which can influence the insertion of the cochlear implant. Various attempts have been made to evaluate the dimensions of the cochlea related to cochlear implantation. Little attention was paid to the distinct narrowing of the scala tympani in the region of the ascending part of the cochlear duct, although from the literature, it is known that electrode insertion trauma frequently occurs here. Individual variations of the cochlear micromorphology may additionally contribute to the failure of preformed electrode arrays, but the challenge of guiding the electrode array around the first bend of the cochlear turn, that is, the pars ascenders, is obviously impaired by the interindividual constant narrowing in this area. Therefore, this finding may have implications for the development of electrode designs and insertion methods. The diameter of the Scala Tympani decreases by approximately 300 microm during the ascending part of the basal turn [2,3]. Thus, in patients with a smaller cochlear base, the insertion of the cochlear electrode is influenced by the appearance of resistance points. The cochlear lumen shows a noncontinuous spiraling path leading to potential pressure points during cochlear implantation at the basilar membrane in the region of 180 to 225 (12–14 mm) and 725 degrees (22–26 mm) and at the floor of the Scala tympani around 0 to 90, 225 to 270, and 405 to 450 degrees [3]. Measuring the basal length of the cochlea before cochlear implantation plays an important role, as it can give valuable indications for anticipating difficulties in electrode insertion [4,5]. From a structural point of view, the cochlear electrode has a diameter of 0.5 mm at the apical level and a diameter of 0.8 mm at the basal level. The full insertion of the cochlear electrode does not exceed the active length of 15 mm [6,7].

**Figure 1:**
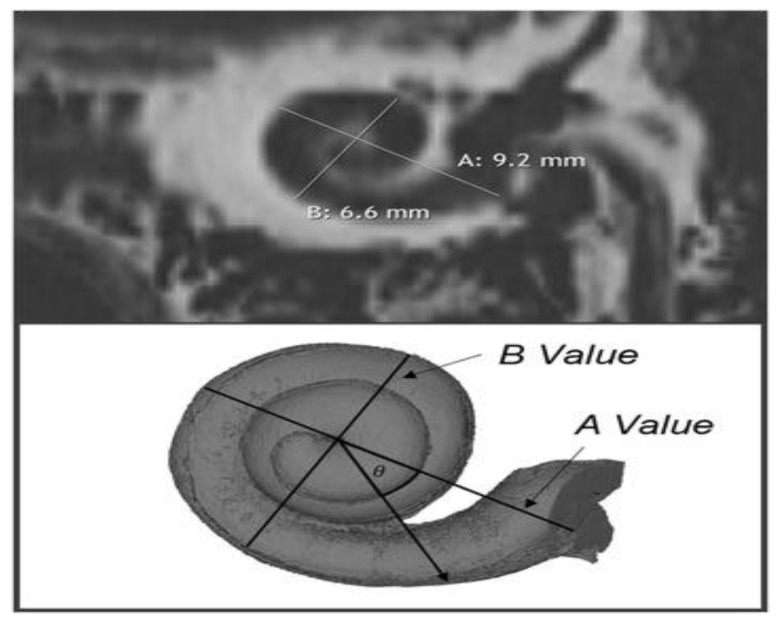
(**A**)—Length and (**B**)—width cochlea measurements.

In general, the complete insertion of the electrode is indicated in order to ensure effective cochlear stimulation and achieve the best hearing performance. However, due to multiple anatomical variations, electrode insertion is difficult to achieve. The preoperative computed tomographic examination is essential not only in assessing the anatomical aspect of the cochlea, but also in determining its dimensions in order to choose an appropriate electrode to obtain the best possible audiological performance. Added to these is the possibility of anticipating some intraoperative complications that could occur [8]. Preoperatively, CT can be helpful in making decisions about the most optimal surgical approach and can help minimize complications during surgery. With the help of computer tomography, bone structures can be properly evaluated, establishing their contours, dimensions, and shapes. The choice of appropriate settings, as well as the use of contrast in the evaluation of the appearance of the cochlea, allow an adequate evaluation of its shape and dimensions. With the help of high-resolution computer tomography, multiplane reconstructions can be made with measurements of the length, width, and basal diameter of the cochlea can be made, the measurement plane being between the round and oval windows in a plane that includes the cochlear canal [9,10], the vestibule, and parts of the semicircular canals (Figure 1).

Knowledge of the cochlear implant array’s precise position is important because of the correlation between electrode position and speech understanding. Several groups have provided recent image processing evidence to determine scalar translocation, angular insertion depth, and cochlear duct length (CDL), all of which are being used for patient-specific programming. Cone beam computed tomography (CBCT) is increasingly used in otology due to its superior resolution and low radiation dose [11]. Measurement of the angular depth of insertion (aDOI) of cochlear implant electrode arrays has numerous clinical and research applications. Plain-film radiographs are easily obtained intraoperatively and have been described as a means to calculate aDOI. CT imaging with 3D reformatting can be used for cochlea measurement but is less conveniently obtained and requires higher radiation doses, a particular concern in pediatrics. The extent to which plain-film and 3D CT image-based measurements are representative of the true position of the electrode within the cochlea is unknown [12]. In the present paper, we aimed to carry out an observational study regarding the role of cochlear measurements in the preoperative evaluation of patients proposed for cochlear implants. The purpose of the study was to measure the cochlea and establish the existence of a correlation between the size of the cochlea and the age and gender of the patients, as well as to show the existence of some of its variations. This study was carried out by using preoperative CT images in implanted patients and evaluating the length and width of the cochlea. The data were correlated with those from the specialized literature as well as with the demographic data of the patients.

It is extremely important to know as accurately as possible the dimensions of the cochlea, from a clinical and surgical point of view. From a clinical point of view, it is important, both from the perspective of post-operative audiological results and a surgical point of view, that the type of electrode must be chosen that correspond to the cochlear dimensions to stimulate as much of the affected cochlea as possible. In this context, this study has clinical applicability, and especially, a practical perspective.

## 2. Materials and Method

This retrospective observational study was performed on a group of 35 patients with bilateral congenital profound sensorineural hearing loss hospitalized in the Targu Mures, Otorhinolaryngologic Department, in the period 2016–2017, where the demographic data regarding the age, sex, and patients were analyzed, and later the data related to the size of the cochlea were added through the analysis of computed tomography images. Computed tomographic investigations were performed using a 128-slice computed tomography with contrast and 0.6 mm sections.

Multiplanar reconstructions with high-resolution CT defined by specific criteria are used to make measurements of the length and width of the cochlea and also of the diameter of the basal lumen. The plane of measurements was determined by an identifiable oval and round window with the cochlear canal, a visible vestibule, and parts of the lateral and superior semicircular canals.

Insertion of the complete cochlear electrode is more difficult to achieve due to cochlear anatomical variations. An accurate preoperative computed tomography (CT) examination could suggest the most appropriate electrode specificities, including operative technique. Minimal damage to the inner ear leads to fewer preoperative complications and easier reimplantation operations.

Axial CT images of the temporal bone highlight the normal basal twisting of the cochlea. The bony spiral lamina is seen as a linear opacity in the center of the basal turn. The entire cochlea can be evaluated by highlighting the interscalar septum between the base and middle peaks. It is thicker than the section between the apical and middle tips. The posterior semicircular canal can be seen at this level. The middle and apical tips of the cochlea are highlighted together with the central part, which is the mediolus. The diaphragm for the cochlear nerve lies between the base of the modiulus and the internal auditory meatus. With Computed Tomography, we have evaluated the vestibule, the semicircular canal, and the vestibular aqueduct, important elements for cochlear implantation.

The criteria for inclusion in the study were taken according to the age of the patients: 0–4 years, 4–7 years, 7–14 years, and over 14 years. The data were analyzed only for patients with profound or severe sensorineural hearing loss, candidates for cochlear implants, being excluded from the study patients with moderate or mild unilateral or bilateral hearing loss, or other types of hearing loss. The collected data were entered and processed in Microsoft Excel tables, and the statistical processing was carried out with the GraphPad InStat3 program, using descriptive statistics. For the comparison of two or more variables, we used the Fischer and student t-tests. To consider a value as statistically significant, we considered a *p* < 0.05. To conduct the study, since we only used computed tomographic evaluations without the involvement of the patients, we obtained the institutional agreement for the use of clinical data.

## 3. Results

**Figure 2:**
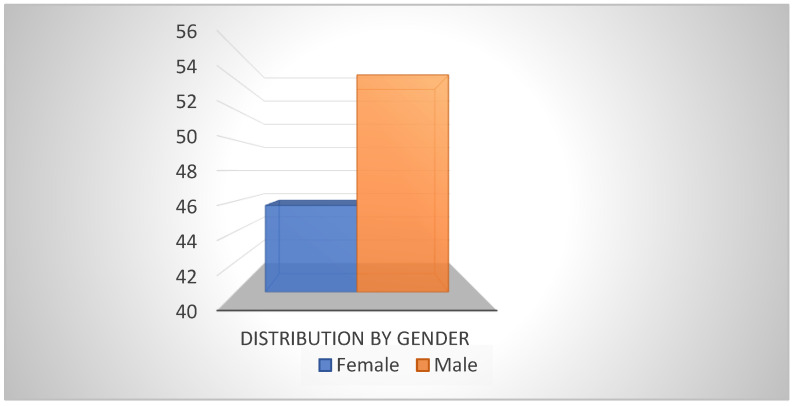
Distribution by gender of patients.

From the group of 35 examined patients, 54% (n = 19) were male and 46% (n = 16) female, which demonstrates in our study the predominance of male patients without this being a general rule of cochlear implantation or a selective criterion for cochlear measurements (Figure 2).

**Figure 3:**
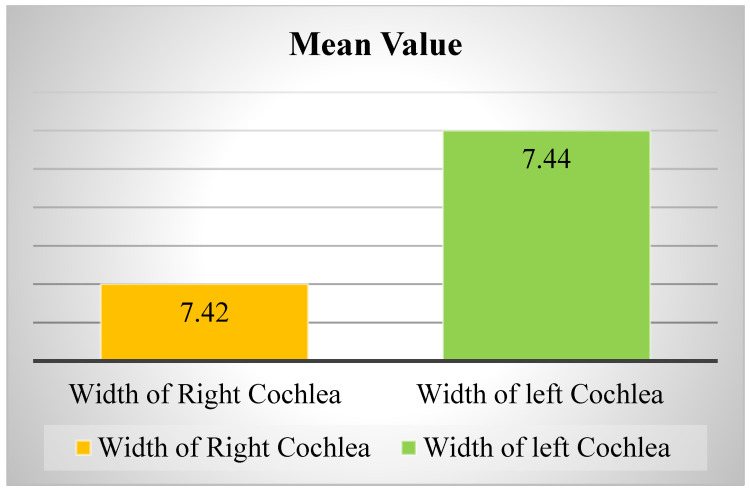

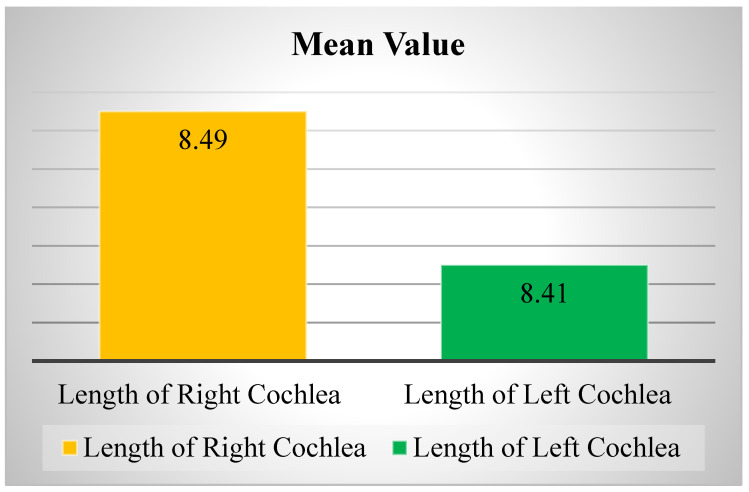
Comparative cochlea dimensions between the left and right ears.

By measuring the width of the cochlea at its base, without taking into account the sex and age of the patients, the presence of an average value of 7.42 ± 2 mm was found without a difference between the two cochleae. The length measurements show the existence of an average value of 8.41 ± 6 mm, without a significant difference between the two cochlea; however, it was found that the width of the cochlea in the right ear is smaller in all examined patients than the left ear, while its length is greater, although the difference is insignificant (Figure 3).

**Figure 4:**
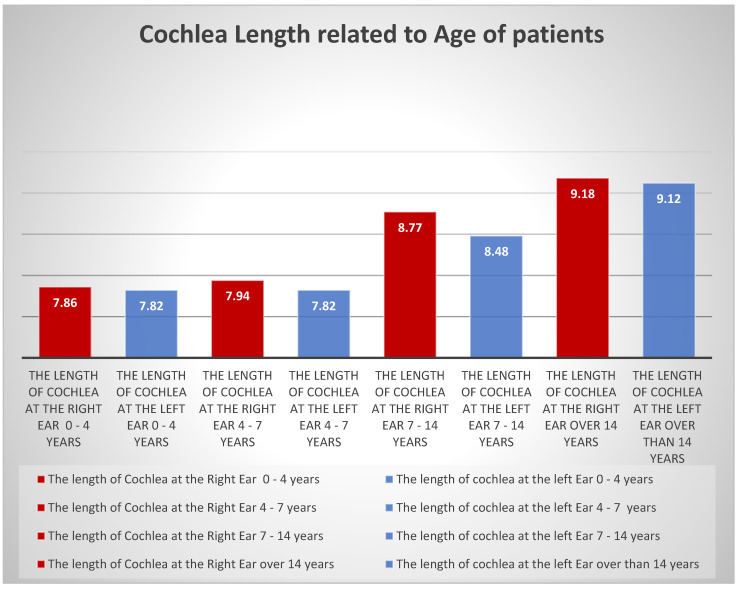
Cochlea length in relation to the age of patients.

Referring to the correlation with the age of the patients, the results were as follows: 8.61 mm for females and 8.41 mm for males, the difference not being significant, taking into account the fact that the measurements have a subjective character. The average length of the cochlea in the age group 0–4 years is 7.82 mm in the left ear and 7.86 mm in the right ear; in the age group 4–7 years, it is 7.82 mm and 7.94 mm, respectively; for the age group 7–14 years, the dimensions increase to 8.48 mm and 8.77 mm, respectively; and after 14 years, these dimensions reach 9.12 mm and 9.18 mm, respectively (Figure 4). Comparative measurements of the length of the cochlea by age groups show an increase in length with the patient’s age, but this increase does not exceed 1.5 mm for both the right and left ears. The measurements of the width of the cochlea, by age group, start from 6.84 mm in the left ear and 6.81 mm in the right ear at 0–4 years; 6.94 mm and 6.97 mm, respectively, at the group 4–7 years; 7.71 mm and 7.55 mm, respectively, at 7–14 years; and reaching 8.19 mm and 8.12 mm, respectively, at the age of over 14 years. In the age group between 0 and 4 years, the optimal age for the cochlear implant, the difference in length between the two ears is 0.04 mm, and the width of the left cochlea is 0.03 mm larger than the right cochlea. In the age group 4–7 years, the difference in length between the two cochlea shows the right cochlea larger than the left cochlea; in contrast, the width measurements demonstrate right cochlea width is 0.03 mm larger than the left cochlea, inverse to the cochlea width measured in the 0–4 year group. The evaluation of the cochlear dimensions in the age group 7–14 years shows first of all a difference of 0.29 mm between the right and left cochlea in length, the right cochlea being larger than the left cochlea—the same as in the age groups 0–4 years and 4–7 years, respectively.

**Figure 5:**
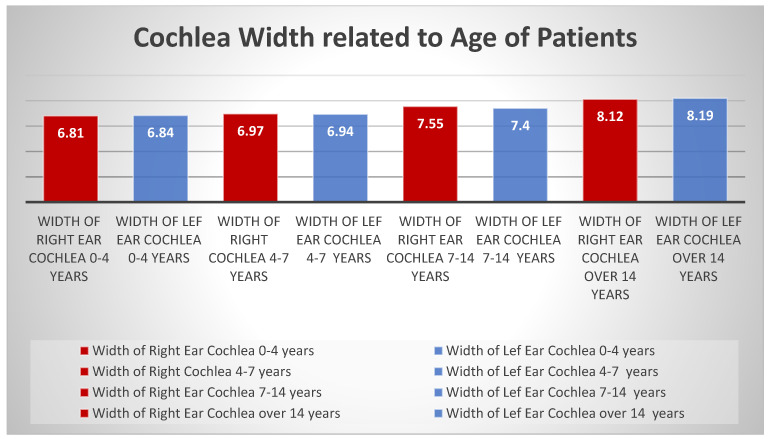
Cochlea width in relation to the age of patients.

The width of the left cochlea is smaller than the right cochlea by 0.15 mm. It is also noticeable here that the left cochlea is smaller than the right, just like in the age group 4–7 years. In the over-14 age group, a difference of 0.06 mm is noted between the lengths of the right and left cochlea, while width measurements show an increase in the size of the left cochlea over the right by 0.07 mm. Comparative measurements of the length of the cochlea by age groups show an increase in length with the patient’s age, but this increase does not exceed 1.5 mm for both the right and left ear. The evaluation of the width of the cochlea shows a gradual increase in it in all age groups for both the right and left cochlea (Figure 5).

As with general measurements, the size difference between the right and left cochlea is maintained. The evaluation of the width of the cochlea shows a gradual increase in it in all age groups for both the right and left cochlea. Correlations between cochlear lengths, cochlear widths, and age groups demonstrate the lack of a specific correlation, with the coefficient being greater than 0.001 (Table 1). 

## 4. Discussion

Cochlear implantation is a proven treatment for severe to profound sensorineural hearing loss. Imaging has an important role in evaluating candidates for implant surgery, providing realistic preoperative counseling, and predicting postoperative outcomes.

Also, imaging investigations provide information on potential difficulties encountered by the doctor during cochlear implantation. High-resolution computed tomography and nuclear magnetic resonance are complementary in evaluating various aspects of the temporal bone and auditory pathways in these patients [13].

The value of high-resolution computed tomography and nuclear magnetic resonance for the routine evaluation of candidates for cochlear implantation varies by patient, although performing both investigations, each with its own costs and risks, is not necessary in every clinical situation. In general, the results of both investigations correlated with the intraoperative anatomical results [10].

According to the specialized literature, nuclear magnetic resonance has a greater sensitivity and specificity in the diagnosis of inner ear abnormalities compared to computed tomography in candidates for cochlear implantation; at the same time, the abnormalities detected with the help of magnetic resonance are more likely to influence the implantation process [13,14].

Also, the identification of abnormalities of the cochlea and modiolus with the help of magnetic resonance was superior to those identified by computed tomography.

However, cochlear measurements on computed tomography provide important data on the size of the cochlea. High-resolution computed tomography and nuclear magnetic resonance are the recommended imaging investigations, either individually or together, as they are complementary in evaluating various aspects of the temporal bone and auditory pathways in these patients [15,16]. Imaging investigations play an important role in the evaluation of cochlear implant candidates, as they help the physician provide realistic preoperative counseling and information on potential difficulties encountered by the physician during cochlear implantation.

Knowledge about cochlear duct length may assist electrode choice in cochlear implantation. However, no gold standard for clinically applicable estimation of cochlear duct length exists. There are studies to determine the most reliable radiological imaging method and imaging processing software for measuring cochlear duct length and other anatomical dimensions from clinical routine imaging and to accurately predict the insertion depth of the CI electrode [15,16].

Computed tomography measures of cochlear distance were used to predict insertion depths, and these were markedly concordant with the actual length of the electrode array required to reach this point [16].

Although in the study carried out by us no significant differences in the growth of the cochlea by age groups were found, neither in the left nor in the right ear; however, knowing the dimensions of the cochlea is essential in the preoperative evaluation of patients for a cochlear implant. From the studied group of 35 patients, the demographic assessment consists of the predominance of the male sex, without this being a general rule for cochlear implantation or a selection criterion for patients. The evaluation of cochlear dimensions by age groups, although statistically insignificant, is nevertheless important from a surgical point of view. The evaluation of age groups shows a prevalence of age 0–4 years, 14 patients; followed by 4–7 years, 5 patients; 7–14 years, 3 patients; and over 14 years, 13 patients. The predominance of patients 0–4 is also an essential criterion for selecting patients for cochlear implants. The evaluation of the length of the cochlea by performing a right-left comparison, showed that there are differences between the two cochlea in all age groups as follows: in the age group between 0 and 4 years, the optimal age for performing the cochlear implant, the difference in length between the two ears is 0.04 mm, and in width, the left cochlea is 0.03 mm larger than the right cochlea. As with general measurements, the size difference between the right and left cochlea is maintained; in the age group 4–7 years, the difference in length between the two cochlea shows the right cochlea being larger than the left cochlea; in contrast, the width measurements demonstrate the right cochlea width being 0.03 mm greater than the left cochlea, which is the inverse of the cochlea width measured in the 0–4 years group; the assessment of cochlear dimensions in the age group 7–14 years shows primarily a difference of 0.29 mm between the right and left cochlea in length, the right cochlea being larger than the left cochlea—the same as in the age groups 0–4 years, respectively 4–7 years. The width of the left cochlea is smaller than the right cochlea by 0.15 mm. It is also noticeable here that the left cochlea is smaller than the right, just like in the age group 4–7 years; in the over 14 age group, there is a difference of 0.06 cm between the lengths of the right and left cochlea, while the width measurements show an increase in the size of the left cochlea compared to the right by 0.07 mm.

Our study can be compared with the studies that evaluate the morphometric measures, which showed that the angular distance between the round window exhibited a narrow range of 684–704°, corresponding to linear distances of 17.87 mm and 34.48 mm along the inner and outer walls of the scala tympani [17].

The first turn measured an average of 14.21 mm along the inner wall and 23.92 mm along the outer wall. The outer wall average for the second turn was 11.11 mm and for the partial third apical turn was only 4.49 mm. The range for cochlear duct angular distance was 876° to 1051°, with a mean of 2.63 turns, corresponding to an average linear distance of 39.53 mm, ranging from 35.44 mm to 43.57 mm. The study demonstrates that the anatomy of the cochlea of CI patients does not differ significantly from that of normative subjects and establishes measurements using the round window as the 0° reference point, an important surgical landmark. The relevance of the measurements to cochlear implant design is discussed [17,18].

The optimal implantation age between 0–4 years is characterized in terms of cochlear dimensions by smaller values in both width and length, but since this difference compared to older ages is not significant, the choice of the type of electrode is not correlated with the patient’s age. There is also a difference between the right and left ears, both in length and width, which may raise the question of whether there is an indication for implants to take this difference into account. The specialized literature shows that cochlear anatomical variations, related to its dimensions, can influence the insertion of the electrode. The transparent cochleae had not undergone any shrinkage or any significant architectural changes. 3D immunofluorescence analysis of the cochlea provided sufficient image resolution for analysis of the spiral ganglion neurons and assessment of the fibrotic tissue reaction surrounding the electrode array. There is a protocol that can offer a microscopical analysis of the cochlea with the implant. This technique is suitable for the study of post-implantation cell and tissue damage in the same sample without the potential toxicity of other methods described to date. This technique can also evaluate the cochlea dimensions with microscopical aspects [18].

## 5. Conclusions

Assessment of cochlear dimensions is essential in preparing patients for cochlear implants. Variables in cochlear dimensions must be taken into account, as there may be anatomical variations of the cochlea, which may influence its size, which influences the way of insertion. Changes in the width of the cochlea are also related to its malformations, influencing the way of insertion when cochleostomy is performed as a way of inserting the electrode. Variations in the dimensions of the cochlea, both in length and width, are related to age and sex, and there is an increase in them with advancing age. This increase is statistically insignificant, but it still exists, even if, from a theoretical point of view, it is considered that the dimensions of the cochlea remain constant. The dimensions change a little, not necessarily related to malformations of the cochlea. The study carried out evaluated the normally conforming cochlea. Knowing the length of the cochlea is important in how to insert the electrode. The explanation is as follows: the values of the length of the cochlea fluctuate according to age and sex, as well as between the right and left cochlea, not significantly, but enough to know it before the implant. The practical applicability consists of the functional necessity of inserting all the electrodes of the implant in order to obtain the expected audiological performances. Audiological performance is related to intraoperative measurements of cochlear implant functionality. These are directly related to the degree of insertion of the electrode into the cochlea, which must be complete, i.e., equivalent to its length of 1.5 mm. The fact that the growth of the cochlea does not occur over time over 1.5 mm, the standard size of the length of the electrode, means that at any age, if there is a normally compliant cochlea, the electrode can be completely inserted into the cochlea, obtaining the expected audiological performances through electrical stimulation.

In the future, there are multiple development possibilities in the field of cochlear measurements, which can achieve a correlation between cochlear structure and function in relation to postoperative results through 3D analysis.

## Figures and Tables

**Table 1:** Comparative results of the cochlear dimensions by age groups of the two ears. ** No significant data.

			Age	Width Left Ear	Width Right Ear
Age	Pearson Correlation		1	0.597 **	0.610 **
	Sig. (2-tailed)			0.000	0.000
	N		35	35	35
Width Left Ear	Pearson Correlation		0.597 **	1	0.963 **
	Sig. (2-tailed)		0.000		0.000
	N		35	35	35
Width Right Ear	Pearson Correlation		0.610 **	0.963 **	1
	Sig. (2-tailed)		0.000	0.000	
	N		35	35	35
			Age	Left Ear length	Right Ear Length
Spearman’s rho	Age	Correlation Coefficient	1.000	0.652 **	0.668 **
		Sig. (2-tailed)	.	0.000	0.000
		N	35	35	35
	Left Ear length	Correlation Coefficient	0.652 **	1.000	0.944 **
		Sig. (2-tailed)	0.000	.	0.000
		N	35	35	35
	Right Ear length	Correlation Coefficient	0.668 **	0.944 **	1.000
		Sig. (2-tailed)	0.000	0.000	.
		N	35	35	35

## Data Availability

Participant-level data are available from the corresponding author.
